# Metabolome Analysis of Constituents in Membrane Vesicles for *Clostridium thermocellum* Growth Stimulation

**DOI:** 10.3390/microorganisms9030593

**Published:** 2021-03-13

**Authors:** Shunsuke Ichikawa, Yoichiro Tsuge, Shuichi Karita

**Affiliations:** 1 Graduate School of Education, Mie University, 1577 Kurimamachiya-cho Tsu, Mie 514-8507, Japan; 2 Faculty of Education, Mie University, 1577 Kurimamachiya-cho Tsu, Mie 514-8507, Japan; 217112@m.mie-u.ac.jp; 3 Graduate School of Bioresources, Mie University, 1577 Kurimamachiya-cho Tsu, Mie 514-8507, Japan; karita@bio.mie-u.ac.jp

**Keywords:** cellulosic biomass utilization, membrane vesicle, cell–cell communication, *Clostridium thermocellum*, *Bacillus subtilis*, metabolome analysis

## Abstract

The cultivation of the cellulolytic bacterium, *Clostridium thermocellum*, can have cost-effective cellulosic biomass utilizations, such as consolidated bioprocessing, simultaneous biological enzyme production and saccharification. However, these processes require a longer cultivation term of approximately 1 week. We demonstrate that constituents of the *C. thermocellum* membrane vesicle fraction significantly promoted the growth rate of *C. thermocellum*. Similarly, cell-free *Bacillus subtilis* broth was able to increase *C. thermocellum* growth rate, while several *B. subtilis* single-gene deletion mutants, e.g., *yxeJ*, *yxeH*, *ahpC*, *yxdK*, *iolF*, decreased the growth stimulation ability. Metabolome analysis revealed signal compounds for cell–cell communication in the *C. thermocellum* membrane vesicle fraction (ethyl 2-decenoate, ethyl 4-decenoate, and 2-dodecenoic acid) and *B. subtilis* broth (nicotinamide, indole-3-carboxaldehyde, urocanic acid, nopaline, and 6-paradol). These findings suggest that the constituents in membrane vesicles from *C. thermocellum* and *B. subtilis* could promote *C. thermocellum* growth, leading to improved efficiency of cellulosic biomass utilization.

## 1. Introduction

Cellulose is one of the most abundant organic materials on Earth. Bacteria that can grow on cellulose have been isolated from many environments that include soil, hot springs, cow rumen, termite gut, and the human intestinal tract [1]. *Clostridium thermocellum* (*Acetivibrio thermocellus*) [2], a Gram-positive thermophilic anaerobic soil bacterium, is a candidate for cellulosic biomass utilization. *C. thermocellum* completely degrades 4.4 g/L purified cellulose in one day [3]. It also degrades 65% of 5 g/L switchgrass in five days and 70% of 10 g/L corn hull in seven days [4,5].

*C. thermocellum* has been shown to produce 1.3% ethanol from 10% Avicel cellulose [6]. A strain of *C. thermocellum* multiply deleted for [FeFe] hydrogenase maturase, lactate dehydrogenase, pyruvate-formate lyase, Pfl-activating enzyme, phosphotransacetylase, and acetate kinase genes, which eliminated formate, acetate, and lactate production, and reduced H_2_ production, presented a titer of 2.2% ethanol from 6% Avicel cellulose [7]. The ethanol hyper-producing strain *C. thermocellum* I-1-B produced 2.4% ethanol from 8% cellulose [8]. A co-culture of a strain lacking the lactate dehydrogenase/phosphotransacetylase gene and *Thermoanaerobacterium saccharolyticum* produced 3.8% ethanol from 9.2% Avicel cellulose in 146 h [9]. These reports show that the cultivation of *C. thermocellum* can be simplified consolidated bioprocessing (CBP). This is a promising strategy because it eliminates the need to add lignocellulose-degrading enzymes that significantly increase the cost of biofuel production [10,11,12].

Some cellulolytic bacteria, including *C. thermocellum*, form carbohydrate-active enzyme (CAZyme) complexes that are termed cellulosomes [13,14,15,16]. The main product of enzymatic cellulose degradation is cellobiose, which leads to the feedback inhibition of cellulosomes. Supplementation with β-glucosidase (BGL) leads to the hydrolysis of cellobiose into form two glucose molecules, thereby resolving the feedback inhibition. *C. thermocellum* preferentially utilizes cellooligosaccharide, and glucose tends to accumulate in the culture broth [17]. Supplementation with purified BGL increased glucose production by *C. thermocellum* from 10% cellulose or 12% alkali pretreated rice straw by approximately 7.7% over 10 days [18]. This technology is referred to as biological simultaneous enzyme production and saccharification (BSES). BSES is similar to CBP, does not require the diverse CAZymes for the saccharification of cellulosic biomass.

We previously reported that *C. thermocellum* produces extracellular membrane vesicles (MVs) that are released into the broth [19]. MVs are produced in Gram-negative and Gram-positive bacteria. The latter possess a membrane that is overlaid by a relatively thick and resilient cell wall enriched in peptidoglycan [20,21]. MVs have been isolated from the culture supernatant of Gram-positive bacteria that include *Bacillus subtilis*, *B. anthracis*, *Streptomyces coelicolor*, *Listeria monocytogenes*, *Staphylococcus aureus*, *Streptococcus mutans*, *S. pneumoniae*, and *Clostridium perfringens* [22,23,24,25,26,27,28]. Klieve et al. reported the production of MVs by *Ruminococcus* spp., a cellulolytic bacterium that resides in the ovine rumen. DNA molecules ranging in size from <20 to 49 kb, and from 23 to 90 kb are attached to MVs from *Ruminococcus* sp. YE73 and *Ruminococcus albus* AR67, respectively. Thus, MVs can function as vectors for horizontal gene transfer to confer cellulolytic activity, as documented in the mutant strain *Ruminococcus* sp. YE71 [29]. MVs from cellulolytic *Bacteroides fragilis* and *B. thetaiotaomicron* are equipped with hydrolytic enzymes and are important in polysaccharide degradation [30,31]. MVs from *Fibrobacter succinogenes* are enriched with CAZymes, and intact MVs are able to degrade a broad range of hemicelluloses and pectin [32]. We have previously proposed that *C. thermocellum* may utilize MVs to deliver cellulosomes, which enhance the cellulolytic activity of *C. thermocellum* [19].

MVs contain various compounds that include DNA and RNA. These cargos are delivered to neighboring cells. MVs have several important functions related to cell–cell interactions. In *Pseudomonas aeruginosa*, a hydrophobic cell–cell communication signal termed *Pseudomonas* quinolone signal is released from the bacteria via MVs [33,34]. MVs can also serve as organic carbon sources for heterotrophs. For example, MVs derived from cyanobacteria support the growth of *Alteromonas* and *Halomonas* as the sole carbon source, indicating that MVs should be considered in the marine food web and may have important roles in the carbon flux of the ocean [35]. In *Mycobacterium tuberculosis*, the causative agent of tuberculosis, increased MV production in response to iron restriction has been observed [36]. These MVs contain a siderophore called mycobactin. Mycobactin can serve as an iron donor to support the growth of iron-starved *M. tuberculosis*.

In this study, we demonstrated that the MV fractions collected from *C. thermocellum* and *B. subtilis* can promote *C. thermocellum* growth. Metabolome analysis was also performed to identify the candidate compounds with the growth stimulation.

## 2. Materials and Methods

### 2.1. Strains and Culture Conditions of C. thermocellum and B. subtilis

One hundred microliters of *C. thermocellum* DSM 1313 (DSMZ, Braunschweig, Germany) culture was inoculated in 5 mL of CTFUD medium (3 g/L sodium citrate tribasic dehydrate, 1.3 g/L (NH_4_)_2_SO_4_, 1.5 g/L KH_2_PO_4_, 130 mg/L CaCl_2_ 2H_2_O, 500 mg/L L-cysteine-HCl, 11.56 g/L 3-morpholinopropanesulfonic acid, 2.6 g/L MgCl_2_ 6H_2_O, 1 mg/L FeSO_4_ 7H_2_O, 4.5 g/L Bacto yeast extract, 1 mg/L resazurin, pH 7.0) containing 0.5% cellobiose (Tokyo Chemical Industry, Tokyo, Japan) with 16 × 125 mm Hungate tubes (Chemiglass Life Sciences, Vineland, NJ, USA), and cultured at 60 °C under anaerobic conditions with nitrogen gas [37].

*B. subtilis* KAO/NAIST chromosomal deletion mutants [38] and BKE genome-scale deletion mutants [39] were obtained from the National BioResource Project *B. subtilis* (National Institute of Genetics, Shizuoka, Japan). *B. subtilis* strains were aerobically cultured in Luria Bertani broth at 37 °C.

### 2.2. Preparation of MV Fraction of C. thermocellum

Five milliliters of *C. thermocellum* and *B. subtilis* culture was centrifuged at 10,000× *g* for 2 min at 4 °C, and the supernatant was filtered through a 0.22-μm syringe filter to remove cells. The filtrate was centrifuged at 179,000× *g* for 1 h at 4 °C and the pellet was washed twice with 2 mL of sterile phosphate-buffered saline (PBS). The pellet was resuspended in PBS and used as the MV fraction. The MV fraction was kept on ice before use.

MVs were visualized using transmission electron microscopy. Six microliter aliquots of the MV fraction was added to 300-mesh carbon and formvar-coated copper grids and incubated for 1 min. After removing the extra solution with filter paper, each specimen was stained with 2% phosphotungstic acid. The sample was observed with a JEM-1011 microscope (JEOL, Tokyo, Japan) at an accelerating voltage of 80 kV.

### 2.3. Growth Evaluation of C. thermocellum with MV Supplementation

One hundred microliters of *C. thermocellum* DSM 1313 culture was inoculated in 5 mL of CTFUD medium containing 0.5% cellobiose with the supplementation of the collected MV fraction. *C. thermocellum* was cultured at 60 °C under anaerobic conditions with nitrogen gas. The *C. thermocellum* growth was evaluated with optical density of the broth at 600 nm.

### 2.4. Liquid Chromatography-Tandem Mass Spectrometry (LC-MS/MS) Analysis of C. thermocellum MV and B. subtilis Broth

The *C. thermocellum* MV fraction was treated with 10 mg/L surfactin, and the filtrate obtained after ultrafiltration with Vivaspin 2-100 K (Cytiva, Marlborough, MA, USA) was used to obtain the constituents in MVs. Cell-free supernatants of *B. subtilis trpC2* and *trpC2 yxeJ* broth were prepared by centrifugation and filtration with a 0.22-μm syringe filter. These specimens were homogenized with zirconia beads in 75% methanol, and the supernatants were collected after centrifugation at 15,000× *g* rpm for 10 min. The supernatants were applied to a MonoSpin C18 column (GL Science, Tokyo, Japan) and were filtered through a 0.22-μm syringe filter.

LC-MS analysis was performed on an Ultimate 3000 rapid separation LC (RSLC) and the Q Exactive system (Thermo Fisher Scientific, Waltham, MA, USA). Ultimate 3000 RSLC analysis was performed with the following parameters: column, InertSustain AQ-C18 (GL Science); column temperature, 40 °C; injection volume, 2 µL; solvent flow rate, 200 µL/min. The eluting solution was 0.1% formic acid containing 2% acetonitrile. The Q Exactive system had the following parameters: measurement time, 3–30 min; ionization method, electrospray ionization; measurement mass range, *m*/*z*: 80–1200; full scan resolution, 70,000; and MS/MS scan resolution, 17,500. The obtained data were analyzed with PowerGetBatch and MFSearcher [40]. The LC-MS analysis was performed in triplicate.

## 3. Results and Discussion

### 3.1. MV Constituents Promote C. thermocellum Growth

**Figure 1:**
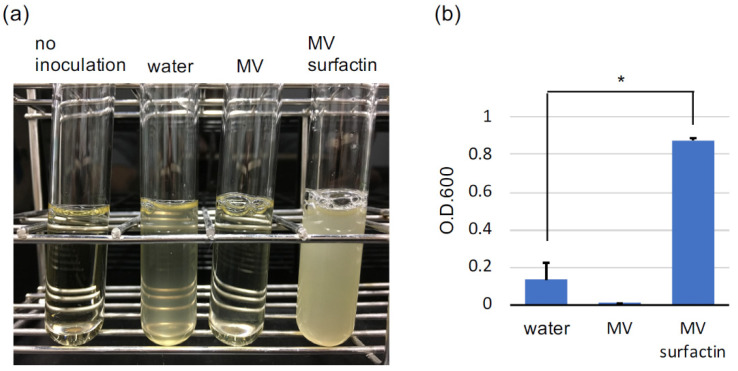
*C. thermocellum* growth stimulation by the MV constituents. *C. thermcellum* was cultured in CTFUD medium for 24 h with the supplementation of water, the MV fraction, or the surfactin-treated MV fraction. The cultures (**a**) and their optical densities (**b**) are shown. The experiment was duplicated. Error bars show standard error. * Student’s *t*-test *p* < 0.01.

A previous study reported that the co-culture of the engineered *C. thermocellum* and *T. saccharolyticum* strains produced 3.8% ethanol from cellulose for 6 days [9]. *C. thermocellum* cultivation with BGL supplementation for 10 days reportedly produced 76.7 g/L glucose from alkali pretreated rice straw [18]. It seems that the growth rate of *C. thermocellum* is an important factor in improving the efficiency of CBP and BSES. In this study, we collected MVs from *C. thermocellum* broth (Figure S1). MVs contain various compounds, such as DNA and RNA, which function in cell–cell communication. When *C. thermocellum* was grown in the presence of the MV fraction, the growth rate did not change. However, when the MVs were lysed using the lipopeptide surfactin [41] the cell density of *C. thermocellum* had significantly increased at 24 h after the inoculation (Figure 1). The surfaction supplementation alone did not affect the *C. thermocellum* growth rate. The final growth yield in each sample had not changed significantly. These results suggest that the constituents in the MV fraction could promote the growth rate of *C. thermocellum*.

### 3.2. B. subtilis Broth Promotes C. thermocellum Growth Rate

**Figure 2:**
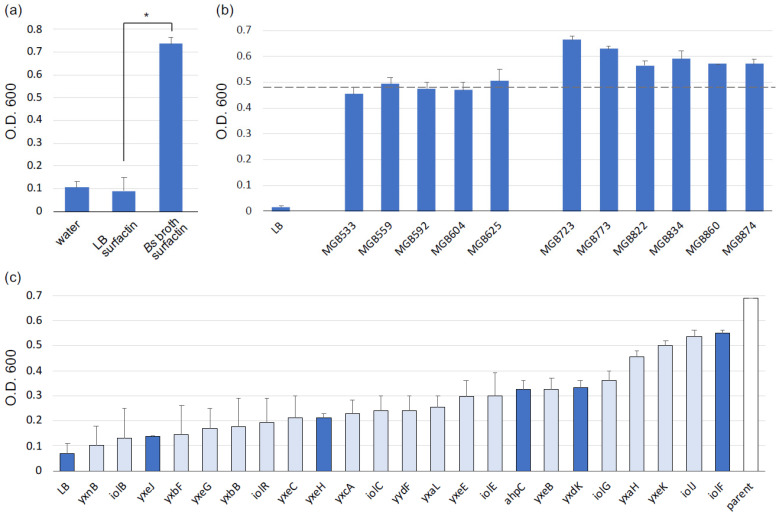
(**a**) *C. thermocellum* growth stimulation using cell-free *B. subtilis* broth. *C. thermcellum* was cultured in CTFUD medium with surfactin-treated cell-free *B. subtilis* broth for 24 h. The experiment was performed in triplicate. * Student’s *t*-test *p* < 0.01. (**b**) *C. thermocellum* growth promotion effect of the broth of *B. subtilis* genome deletion mutants evaluated. The genotypes of the genome deletion mutants are listed in Table S1. The experiment was duplicated. (**c**) Evaluation of the *C. thermocellum* growth promotion effect of the broth of *B. subtilis* single-gene deletion mutants. Dark and light blue indicate significant differences compared with the effect of the parent strain (*trpC2*) with Student’s *t*-test at *p* < 0.01 and < 0.05, respectively. The experiment was duplicated. Error bars indicate the standard error.

Cell-free *B. subtilis* broth containing MVs also promoted the *C. thermocellum* growth rate, similar to the *C. thermocellum* MV fraction (Figure S1 and Figure 2a). Again, the surfaction supplementation alone did not affect the *C. thermocellum* growth rate (Figure 2a). Mukamolova et al. purified the resuscitation promoting factor (Rpf) from the broth of the Gram-positive bacterium, *Micrococcus luteus*. The purified Rpf promoted the growth of this bacterium as well as *Mycobacterium avium*, *M. bovis*, *M. kansasii*, *M. smegmatis*, and *M. tuberculosis* [42]. Genes homologous to the *rpf* gene were found to be widespread in a number of *Mycobacterium* species, as well as in Gram-positive bacteria with a high GC content, such as *Corynebacterium gultamicum* and *Streptomyces rimosus*. The Rpf protein shows peptidoglycan degradation activity [43]. Shah et al. reported that muropeptide fragments released from the peptidoglycan of the Gram-positive bacterium, *B. subtilis*, stimulate the germination of bacterial spores. Staurosporine, which inhibits related eukaryotic kinases in bacteria, blocks muropeptide-dependent bacterial spore germination [44]. We evaluated the effect of staurosporine on *C. thermocellum* growth with cell-free *B. subtilis* broth, however no significant inhibition was observed.

We further evaluated the *C. thermocellum* growth promotion effect of the broth of *B. subtilis* genome deletion mutants [38]. All the mutants, especially six mutants in which the *pdp-rocR* genomic region, were deleted (MGB723, MGB773, MGB822, MGB834, MGB860, MGB874) promoted *C. thermocellum* growth by accelerating the growth rate (Figure 2b, Table S1). Subsequently, we evaluated the *C. thermocellum* growth promotion effect of 100 *B. subtilis* mutants in which single genes within the *pdp-rocR* genomic region were deleted under a *trpC2* gene deletion background (Table S2) [39]. We did not find *B. subtilis* mutants that promoted *C. thermocellum* growth more than *trpC2* strain as the parent strain. Contrary to our expectation, the effect of 23 *B. subtilis* mutants was significantly lower than that of the parent strain (Figure 2c).

Among these 23 genes, the functions of several genes have been experimentally evaluated. The *asnH* operon, which comprises *yxbB*, *yxbA*, *yxnB*, *asnH*, and *yxaM*, might be involved in the biosynthesis of asparagine [45]. The *iolJ*, *iolG*, *iolF*, *iolE*, *iolC*, *iolB*, and *iolR* genes in the *iolABCDEFGHIJ* and *iolRS* operon are responsible for *myo*-inositol catabolism involving multiple and stepwise reactions [46,47,48]. We observed a slight growth inhibition of *C. thermocellum* in the presence of *myo*-inositol, however this required a high concentration (1 mg/mL) of *myo*-inositol (Figure S2). YydF is predicted to be an exported and modified peptide that has antimicrobial and/or signaling properties [49,50]. YxaL, which contains a repeated pyrrolo-quinoline quinone (PQQ) domain that forms a beta-propeller structure, interacts with the DNA helicase PcrA in *B. subtilis* [51]. Kim et al. reported that treatment of *Arabidopsis thaliana* and *Oryza sativa* L. seeds with 1 mg/L purified YxaL was effective in improving root growth [52]. PQQ, which was first recognized as an enzyme cofactor in bacteria, displays bioactivities for various eukaryotes and prokaryotes. For many bacterial species, PQQ has growth stimulation effect and serves as a cofactor for a special class of dehydrogenases/oxidoreductases [53]. PQQ has been described as an essential growth factor for various microbes [54,55,56]. We observed a slight *C. thermocellum* growth promotion effect by PQQ. This effect was not enough to explain the effect of *B. subtilis* broth (Figure S3). More than 50 proteins are involved in *B. subtilis* spore coat assembly. Of these, YxeE is an inner spore coat protein [57,58]. *ahpC* encodes thiol-specific peroxidase that plays a role in protecting cells against oxidative stress by detoxifying peroxides [59]. Utilization of a hydroxamate siderophore, ferrioxamine, requires the FhuBGC ABC transporter together with a ferrioxamine-binding protein, YxeB [60]. A range of siderophores can act as growth factors for various previously uncultured bacteria [61]. YxdK is assumed to be a subunit of the two-component sensor histidine kinase, with its potential cognate response regulator, YxdJ [62]. Co-cultivation with *B. subtilis* allows the growth of *Synechococcus leopoliensis* CCAP1405/1 on solid media. However, the *yxdK* deletion mutant reportedly loses this ability [63]. The *yxeK* gene, which encodes FAD-dependent monooxygenase, contributes to the metabolism of S-(2-succino)cysteine to cysteine [64].

### 3.3. Metabolome Analysis of the Constituents in C. thermocellum MV and B. subtilis Broth

We collected the constituents in *C. thermocellum* MVs and analyzed them using LC-MS/MS. Among the 534 detected peaks, the intensities of seven peaks were significantly higher in the fraction where MVs had been disrupted by surfactin compared to MVs not disrupted using surfactin (Table S3). The structure of five significantly detected compounds in surfactin-treated *C. thermocellum* MVs specimen can be estimated by MS/MS analysis (Table 1 and Table S5).

An aliphatic compound with the chemical formula C_12_H_22_O_2_ was specifically detected in surfactin-treated *C. thermocellum* MVs (Table 1). Cis-2-decenoic acid was reported to decrease persister formation and revert dormant cells to a metabolically active state. Wang et al. demonstrated that three medium-chain unsaturated fatty acid ethyl esters (ethyl trans-2-decenoate, ethyl trans-2-octenoate, and ethyl cis-4-decenoate) decreased persister formation in *Escherichia coli*, *P. aeruginosa*, and *Serratia marcescens*, suggesting that fatty acid ethyl esters disrupt bacterial dormancy [65].

Some aliphatic acids function as diffusible signal factors (DSFs). These include cis-11-methyl-2-dodecenoic acid from *Xanthomonas campestris* and cis-2-dodecenoic acid from *Burkholderia cenocepacia*, among others [66]. DSFs are synthesized by and interact with a diverse group of microbes, including fungi, suggesting a broad conservation of cell-cell communication among these organisms [67,68,69,70]. Mutation of the DSF biosynthesis gene in *B. cenocepacia* results in substantially impaired growth in minimal medium [71]. Dean et al. demonstrated that *Burkholderia* DSF inhibits the formation and disperses *Francisella* biofilms. Furthermore, *Burkholderia* DSF was reported to upregulate the genes involved in iron acquisition in *F. novicida*, which increased siderophore production [72].

Subsequently, we compared the metabolites in the broth of *B. subtilis trpC2* and *trpC2 yxeJ* (Figure 2). Among the 3150 detected peaks, the intensities of 40 peaks were significantly higher in the broth of *B. subtilis trpC2* compared to that of *trpC2 yxeJ* (Table S4). The structures of 32 significantly detected compounds in *B. subtilis trpC2* broth were estimated by MS/MS analysis (Table 2 and Table S5). Diverse peptides were detected in *B. subtilis trpC2* broth. Nicotinamide reportedly enhances growth of both Gram-negative and Gram-positive bacteria, such as *M. avium*, *Propionibacterium acnes*, *S. aureus*, and *B. macerans* [73,74,75,76]. Indole-3-carboxaldehyde was shown to efficiently inhibit biofilm formation by *Vibrio cholerae* O1 [77]. The utilization of urocanic acid by *Pseudomonas* and *Aeromonas* strains has been reported [78,79]. Nopaline is a carbon and nitrogen source metabolized by *Agrobacterium*. 6-Paradol was reported to have significant anti-adhesive activity against *S. aureus* [80].

In this study, we demonstrated that constituents in membrane vesicles significantly promoted the growth rate of *C. thermocellum*. Additionally, the MV constituents with growth stimulation were described by LC-MS/MS analysis. These findings suggest that the constituents in membrane vesicles could promote *C. thermocellum* growth, leading to improved efficiency of cellulosic biomass utilization.

## Figures and Tables

**Table 1:** The constituents in *C. thermocellum* MVs detected by LC-MS/MS analysis.

No.	Formula	Exact Mass	Name	Database	Database ID
3203	C_16_H_31_O_1_N_1_	253.241		EX-HR2	
3013	C_12_H_22_O_2_	198.162	Ethyl 2-decenoate	UC2	HMDB0037329
	C_12_H_22_O_2_	198.162	Ethyl 4-decenoate	UC2	HMDB0039220
	C_12_H_22_O_2_	198.162	Methyl 9-undecenoate	UC2	HMDB0037305
	C_12_H_22_O_2_	198.162	Methyl 10-undecenoate	UC2	HMDB0029585
	C_12_H_22_O_2_	198.162	Allyl nonanoate	UC2	HMDB0029763
	C_12_H_22_O_2_	198.162	cis-3-Hexenyl hexanoate	UC2	HMDB0033378
	C_12_H_22_O_2_	198.162	2-Hexenyl hexanoate	UC2	HMDB0038924
	C_12_H_22_O_2_	198.162	Hexyl 2E-hexenoate	UC2	HMDB0038269
	C_12_H_22_O_2_	198.162	Hexyl 2-methyl-3-pentenoate	UC2	HMDB0040158
	C_12_H_22_O_2_	198.162	Hexyl 2-methyl-4-pentenoate	UC2	HMDB0040163
	C_12_H_22_O_2_	198.162	1-Ethenylhexyl butanoate	UC2	HMDB0037498
	C_12_H_22_O_2_	198.162	2-Octenyl butyrate	UC2	HMDB0038081
	C_12_H_22_O_2_	198.162	cis-4-Decenyl acetate	UC2	HMDB0032214
	C_12_H_22_O_2_	198.162	Menthyl acetate	UC2	C00036314
	C_12_H_22_O_2_	198.162	Rhodinyl acetate	UC2	HMDB0037186
	C_12_H_22_O_2_	198.162	Citronellyl acetate	UC2	C00035564
	C_12_H_22_O_2_	198.162	2-Dodecenoic acid	UC2	HMDB0010729
	C_12_H_22_O_2_	198.162	4-dodecenoic acid	UC2	C00051284
	C_12_H_22_O_2_	198.162	5-dodecenoic acid	UC2	HMDB0000529
	C_12_H_22_O_2_	198.162	11-Dodecenoic acid	UC2	HMDB0032248
	C_12_H_22_O_2_	198.162	5-dodecalactone	UC2	HMDB0037742
	C_12_H_22_O_2_	198.162	gamma-Dodecalactone	UC2	C00030347
	C_12_H_22_O_2_	198.162	epsilon-Dodecalactone	UC2	HMDB0038895
	C_12_H_22_O_2_	198.162	alpha-Heptyl-gamma-valerolactone	UC2	HMDB0037813
	C_12_H_22_O_2_	198.162	4-butyl-4-hydroxyoctanoic acid lactone	UC2	HMDB0036182
	C_12_H_22_O_2_	198.162	2,6-Dimethyl-5-heptenal propyleneglycol acetal	UC2	HMDB0032235
	C_12_H_22_O_2_	198.162	citral dimethyl acetal	UC2	HMDB0040361
	C_12_H_22_O_2_	198.162	citronelloxyacetaldehyde	UC2	HMDB0041449
	C_12_H_22_O_2_	198.162	Chokol A	UC2	C00011518
	C_12_H_22_O_2_	198.162	Cybullol	UC2	C00013221
42	C_12_H_4_O_3_S_1_	227.988		EX-HR2	
3000	C_18_H_35_O_2_N	297.267	Lepadin D	UC2	C00026353
	C_18_H_35_O_2_N	297.267	Cassine	UC2	C00002027
2834	C_8_H_13_ON	139.100	5-Pentyloxazole	UC2	HMDB0038792
	C_8_H_13_ON	139.100	4,5-Dimethyl-2-propyloxazole	UC2	HMDB0037869
	C_8_H_13_ON	139.100	4,5-Dimethyl-2-isopropyloxazole	UC2	HMDB0037871
	C_8_H_13_ON	139.100	4-Butyl-2-methyloxazole	UC2	HMDB0037855
	C_8_H_13_ON	139.100	2,4-Dimethyl-5-propyloxazole	UC2	HMDB0037868
	C_8_H_13_ON	139.100	4,5-Diethyl-2-methyloxazole	UC2	HMDB0037870
	C_8_H_13_ON	139.100	2-Pentyloxazole	UC2	HMDB0037818
	C_8_H_13_ON	139.100	7beta-Hydroxy-1-methylene-8alpha-pyrrolizidine	UC2	C00026172
	C_8_H_13_ON	139.100	2-propionyltetrahydropyridine	UC2	HMDB0034884
	C_8_H_13_ON	139.100	alpha-Phosphinylbenzyl alcohol	UC2	HMDB0029613
	C_8_H_13_ON	139.100	Supinidine	UC2	C00002120
	C_8_H_13_ON	139.100	Tropinone	UC2	C00037960

**Table 2:** The constituents in *B. subtilis trpC2* broth detected by LC-MS/MS analysis.

No.	Formula	Exact Mass	Name	Database	Database ID
1938	C_12_H_23_O_8_N	309.142	4-O-beta-D-Glucopyranosylfagomine	UC2	C00049954
1980	C_15_H_39_O_2_N_7_S_3_	445.233		EX-HR2	
453	C_16_H_30_O_6_N_6_	402.223		EX-HR2	
2242	C_17_H_31_O_4_N_3_	341.231	Diprotin A	UC2	C00018579
1607	C_25_H_40_O_7_	452.277	Briarellin P	UC2	C00044586
799	C_10_H_20_O_3_N_2_S	248.119	Valyl-Methionine	UC2	HMDB0029133
	C_10_H_20_O_3_N_2_S	248.119	Methionyl-Valine	UC2	HMDB0028986
510	C_11_H_22_O_4_N_4_	274.164	Glutaminyllysine	UC2	HMDB0028802
	C_11_H_22_O_4_N_4_	274.164	Lysyl-Gamma-glutamate	UC2	HMDB0028965
	C_11_H_22_O_4_N_4_	274.164	Lysyl-Glutamine	UC2	HMDB0028949
960	C_21_H_40_O_1_N_3_P_3_	443.238		EX-HR2	
2575	C_8_H_13_N_3_P_2_	213.058		EX-HR2	
2345	C_19_H_29_N_3_O_4_S_1_	395.188	V1M1F1	Pep1000	
2536	C_29_H_36_O_5_N_4_	520.269	Lotusine F	UC2	C00027221
	C_29_H_36_O_5_N_4_	520.269	Nummularine S	UC2	C00029150
2237	C_33_H_44_O_11_	616.288	Neoazedarachin A	UC2	C00039833
	C_33_H_44_O_11_	616.288	YM 47524	UC2	C00016365
2633	C_23_H_55_O_12_N_1_P_2_	599.320		EX-HR2	
2673	C_46_H_67_O_2_N_10_P_1_S_1_	854.491		EX-HR2	
1271	C_27_H_44_O_9_	512.299	Butyrolactol B	UC2	C00016754
	C_27_H_44_O_9_	512.299	Integristerone B	UC2	C00048431
	C_27_H_44_O_9_	512.299	Platenolide B mycarose	UC2	C00018288
162	C_6_H_6_ON_2_	122.048	Nicotinamide	UC2	C00000209
	C_6_H_6_ON_2_	122.048	2-Acetylpyrazine	UC2	HMDB0031861
1710	C_15_H_24_O_4_N_4_	324.180		EX-HR2	
211	C_6_H_9_O_3_N	143.058	SQ 26517	UC2	C00018434
	C_6_H_9_O_3_N	143.058	Trimethadione	UC2	HMDB0014491
	C_6_H_9_O_3_N	143.058	6-Oxopiperidine-2-carboxylic acid	UC2	HMDB0061705
	C_6_H_9_O_3_N	143.058	5-ethyl-5-methyl-2,4-oxazolidinedione	UC2	HMDB0061082
	C_6_H_9_O_3_N	143.058	Vinylacetylglycine	UC2	HMDB0000894
	C_6_H_9_O_3_N	143.058	Methyl pyroglutamate	UC2	C00051578
1258	C_22_H_66_N_2_P_2_S_6_	612.303		EX-HR2	
994	C_20_H_33_N_5_O_8_	471.233	G2[L|I]1E1P1, G1A1V1E1P1, G1A1[L|I]1D1P1, G1T2P2, A2V1D1P1, A1S1T1P2, V1E1Q1P1, [L|I]1D1Q1P1, [L|I]1E1N1P1	Pep1000	
655	C_16_H_27_N_5_O_6_	385.196	G3V1P1, G1A3P1, G1V1N1P1, A2Q1P1	Pep1000	
1034	C_10_H_16_O_3_N_2_	212.116	Butabarbital	UC2	HMDB0014382
	C_10_H_16_O_3_N_2_	212.116	L-prolyl-L-proline	UC2	HMDB0011180
	C_10_H_16_O_3_N_2_	212.116	Butethal	UC2	HMDB0015442
457	C_32_H_48_O_5_N_2_S_1_	572.328		EX-HR2	
2755	C_9_H_7_ON	145.053	Indole-3-carboxaldehyde	UC2	C00000112
	C_9_H_7_ON	145.053	2-Quinolone	UC2	C00044432
2680	C_67_H_108_O_6_N_2_S_5_	1196.681		EX-HR2	
115	C_6_H_6_O_2_N_2_	138.043	4-Methoxylonchocarpin	UC2	HMDB0031338
	C_6_H_6_O_2_N_2_	138.043	2-Aminonicotinic acid	UC2	HMDB0061680
	C_6_H_6_O_2_N_2_	138.043	Urocanic acid	UC2	HMDB0062562
	C_6_H_6_O_2_N_2_	138.043	Nicotinamide N-oxide	UC2	HMDB0002730
2949	C_11_H_21_ON	183.162	Tecostanin	UC2	C00001984
	C_11_H_21_ON	183.162	Incarvilline	UC2	C00050294
1600	C_20_H_57_O_4_N_9_S_3_	583.370		EX-HR2	
1727	C_11_H_20_O_6_N_4_	304.138	Nopaline	UC2	C00001548
526	C_21_H_56_O_14_N_10_P_2_	734.345		EX-HR2	
3061	C_17_H_26_O_3_	278.188	1-Acetoxy-3,15-epoxygymnomitrane	UC2	C00021889
	C_17_H_26_O_3_	278.188	Litsealactone B	UC2	C00044889
	C_17_H_26_O_3_	278.188	9beta-Acetoxy-10(14)-aromadendren-4beta-ol	UC2	C00021235
	C_17_H_26_O_3_	278.188	Furoscrobiculin C	UC2	C00021531
	C_17_H_26_O_3_	278.188	[S-[R *,S *-(E)]]-6-[6-(Acetyloxy)-1,5-dimethyl-4-hexenyl]-3-methyl-2-cyclohexen-1-one	UC2	C00011679
	C_17_H_26_O_3_	278.188	Panaxytriol	UC2	C00030923
	C_17_H_26_O_3_	278.188	Panaxacol	UC2	HMDB0039251
	C_17_H_26_O_3_	278.188	Parahigginol C	UC2	C00049252
	C_17_H_26_O_3_	278.188	[1S-(1R *,2E,4R *,5R *,6E,10R *)]-3, 7, 11, 11-Tetramethylbicyclo [8.1.0]undeca-2,6-diene-4,5-diol 5-acetate	UC2	C00012427
	C_17_H_26_O_3_	278.188	Isoobtusilactone	UC2	C00050966
	C_17_H_26_O_3_	278.188	8beta-Acetoxy-9beta-hydroxyverboccidenten	UC2	C00020229
	C_17_H_26_O_3_	278.188	Lincomolide B	UC2	C00047968
	C_17_H_26_O_3_	278.188	[1S-(1R *,2E,4R *,5R *,6E,10R *)]-3, 7, 11, 11-Tetramethylbicyclo[8.1.0]undeca-2,6-diene-4,5-diol 4-acetate	UC2	C00012428
	C_17_H_26_O_3_	278.188	4alpha-Hydroxygymnomitryl acetate	UC2	C00021894
	C_17_H_26_O_3_	278.188	4-[(4E)-3-hydroxydec-4-en-1-yl]-2-methoxyphenol	UC2	HMDB0137260
	C_17_H_26_O_3_	278.188	Ro 09-1544	UC2	C00017230
	C_17_H_26_O_3_	278.188	6-Paradol	UC2	C00002764
	C_17_H_26_O_3_	278.188	Paralemnolin D	UC2	C00030924
	C_17_H_26_O_3_	278.188	Fenoksan; Fenoxan; Fenozan; Fenozan acid; Irganox 1310; Phenosan; Phenoxan; Phenozan	UC2	C00016759
	C_17_H_26_O_3_	278.188	[4aR-(4aalpha,5alpha,8abeta,9abeta)]-9a-Ethoxy-4a, 5, 6, 7, 8, 8a, 9, 9a-octahydro-3,4a,5-trimethyl-naphtho[2,3-b]furan-2(4H)-one	UC2	C00017405
	C_17_H_26_O_3_	278.188	Petasipalin B	UC2	C00020246
	C_17_H_26_O_3_	278.188	4-epi-7alpha,15-dihydroxypodocarp-8(14)-en-13-one;(-)-4-epi-7alpha,15-dihydroxypodocarp-8(14)-en-13-one	UC2	C00035020
	C_17_H_26_O_3_	278.188	3-[(Acetyloxy)methyl]-6-(1,5-dimethyl-4-hexenyl)-2-cyclohexen-1-one	UC2	C00011682
	C_17_H_26_O_3_	278.188	Cyclokessyl acetate	UC2	C00020354
	C_17_H_26_O_3_	278.188	8-Acetoxy-4-acoren-3-one	UC2	HMDB0030974
832	C_12_H_34_O_3_N_6_S_3_	406.185		EX-HR2	

## Data Availability

The data presented in this study are available in insert article or supplementary material here.

## Supplementary Materials

The following are available online at https://www.mdpi.com/2076-2607/9/3/593/s1, Figure S1: MVs from *C. thermocellum* and *B. subtilis*. Figure S2: Effect of *myo*-inositol on *C. thermocellum* growth. Figure S3: Effect of pyrrolo-quinoline quinone on *C. thermocellum* growth. Table S1: Genotypes of *B. subtilis* genome deletion mutants. Table S2: *B. subtilis* single gene deletion mutants used in this study. Table S3: Intensities of detected peaks in the MV fraction of *C. thermocellum* by LC-MS/MS. Table S4: Intensities of the detected peaks in cell-free *B. subtilis trpC2* broth by LC-MS/MS. Table S5: Structures of constituents detected by LC-MS/MS in this study.
