# Supplementary material for: Metabolome Analysis of Constituents in Membrane Vesicles for Clostridium thermocellum Growth Stimulation
Source: Microorganisms. 2021 Mar 13;9(3):593. doi: 10.3390/microorganisms9030593 (PMC8002186; doi:10.3390/microorganisms9030593)
Supplement: Supplementary file 1 [file microorganisms-09-00593-s001.zip › 210206 Supplementary file /210206 legends_for_supplemantal_data.docx]

Fig. S1. MVs from *C. thermocellum* and *B. subtilis.* MVs were collected from *C. thermocellum* (A, C) and from *B. subtilis* (B, D) culture supernatants by ultracentrifugation. The MVs were stained with 2% phosphotungstic acid and visualized by transmission electron microscopy.

Fig. S2. Effect of *myo-*inositol on *C. thermocellum* growth. *C. thermocellum* was cultured in CTFUD medium with *myo-*inositol for 26.5 h. The horizontal axis indicates the concentration of *myo-*inositol. *myo*-Inositol (1 mg/ml) inhibited *C. thermocellum* growth. The experiment was performed in triplicate. Error bars indicate the standard error. *Student’s t-test p < 0.05.

Fig. S3. Effect of pyrrolo-quinoline quinone on *C. thermocellum* growth. *C. thermocellum* was cultured in CTFUD medium with pyrrolo-quinoline quinone for 24 h. The experiment was performed in triplicate. Error bars indicate the standard error.

Table S1. Genotypes of *B. subtilis* genome deletion mutants. The genotypes of *B. subtilis* genome deletion mutants used in this study are listed (Morimoto *et al*. 2008. DNA Research 15: 83–91). Cell-free broths of these *B. subtilis* mutants were utilized for the *C. thermocellum* growth stimulation assay (Fig. 2B).

Table S2. *B. subtilis* single gene deletion mutants used in this study. Cell-free broths of the *B. subtilis* single gene deletion mutants were used for *C. thermocellum* the growth stimulation assay (Koo *et al*. 2017. Cell Systems 4: 291-305). The growth stimulation effect of mutants, indicated by the orange color, was significantly decreased compared to that of the parent strain (Fig. 2C). The descriptions of gene function are from the National BioResource Project *Bacillus subtilis* (https://shigen.nig.ac.jp/bsub/gene/list).

Table S3. Intensities of detected peaks in the MV fraction of *C. thermocellum* by LC-MS/MS. The *C. thermocellum* MV fraction was treated with 10 mg/L surfactin, and filtrate after ultrafiltration with Vivaspin 2-100K (Cytiva, MA, USA) was corrected to obtain the MV constituents. The constituents were analyzed by LC-MS/MS, and the obtained data were compared to those obtained without surfactin treatment. The intensities of the detected peaks are listed. The intensities of the detected peaks highlighted with orange color were significantly (Student’s t-test, p < 0.01) higher in the specimen treated with surfactin. The experiment was performed in triplicate.

Table S4. Intensities of the detected peaks in cell-free *B. subtilis trpC2* broth by LC-MS/MS. The metabolites in the broth of *B. subtilis trpC2* and *trpC2 yxeJ* were analyzed by LC-MS/MS. The intensities of the detected peaks are listed. The intensities of the detected peaks highlighted with orange color were significantly (Student’s t-test, p < 0.01) higher in *B. subtilis trpC2* broth than the level in *trpC2 yxeJ*. The experiment was performed in triplicate.

Table S5. Structures of constituents detected by LC-MS/MS in this study. The structure of detected constituents was estimated by MS/MS analysis. From the obtained exact mass, the chemical formulae were searched using the UC2, EX-HR2, and Pep1000 databases and PowerGetBatch and MFSearcher (Sakurai *et al*. 2013. Bioinformatics 29: 290–91).
