# Supplementary figures and images for: Metabolome Analysis of Constituents in Membrane Vesicles for Clostridium thermocellum Growth Stimulation
Source: Microorganisms. 2021 Mar 13;9(3):593. doi: 10.3390/microorganisms9030593 (PMC8002186; doi:10.3390/microorganisms9030593)

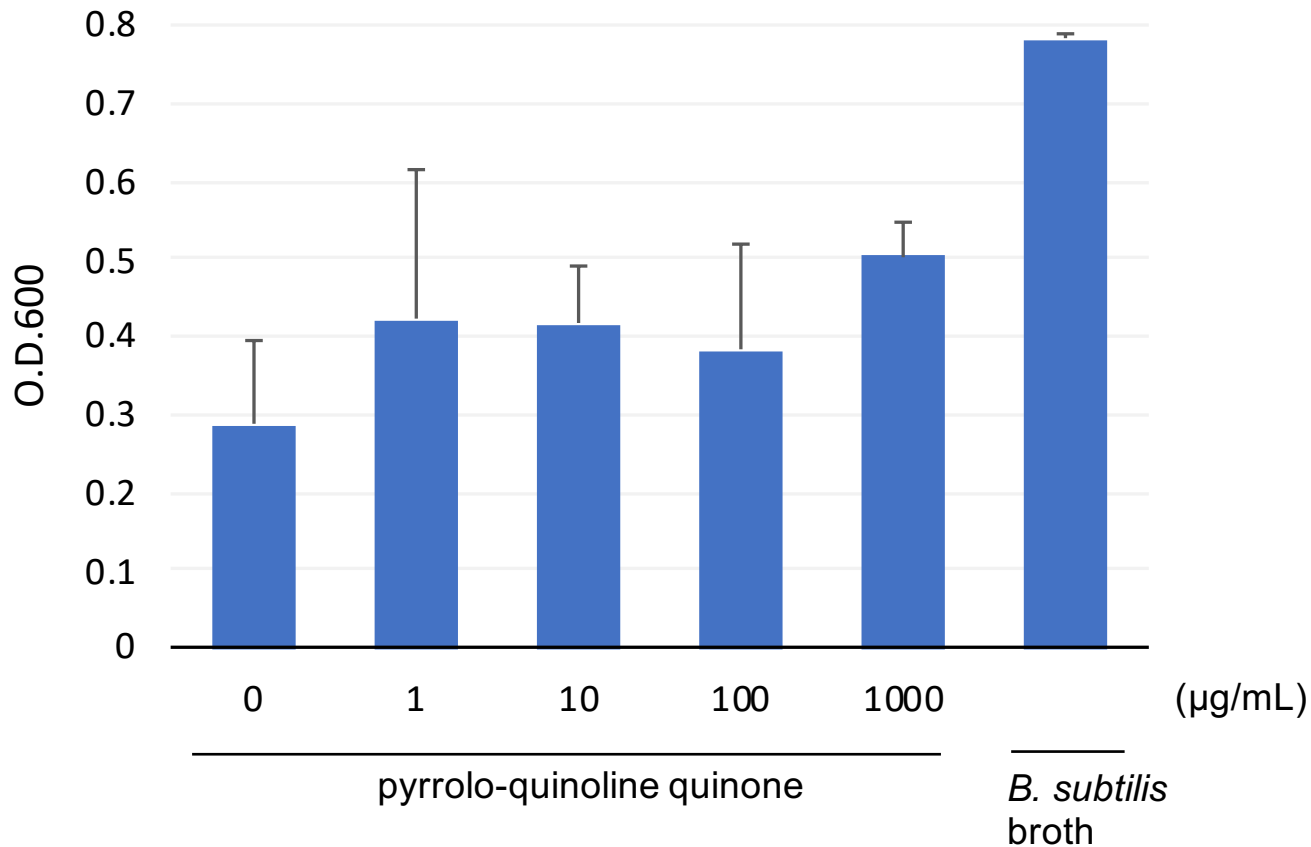

Supplement: Supplementary file 1 [file microorganisms-09-00593-s001.zip › 210206 Supplementary file /Fig.S3.pdf]

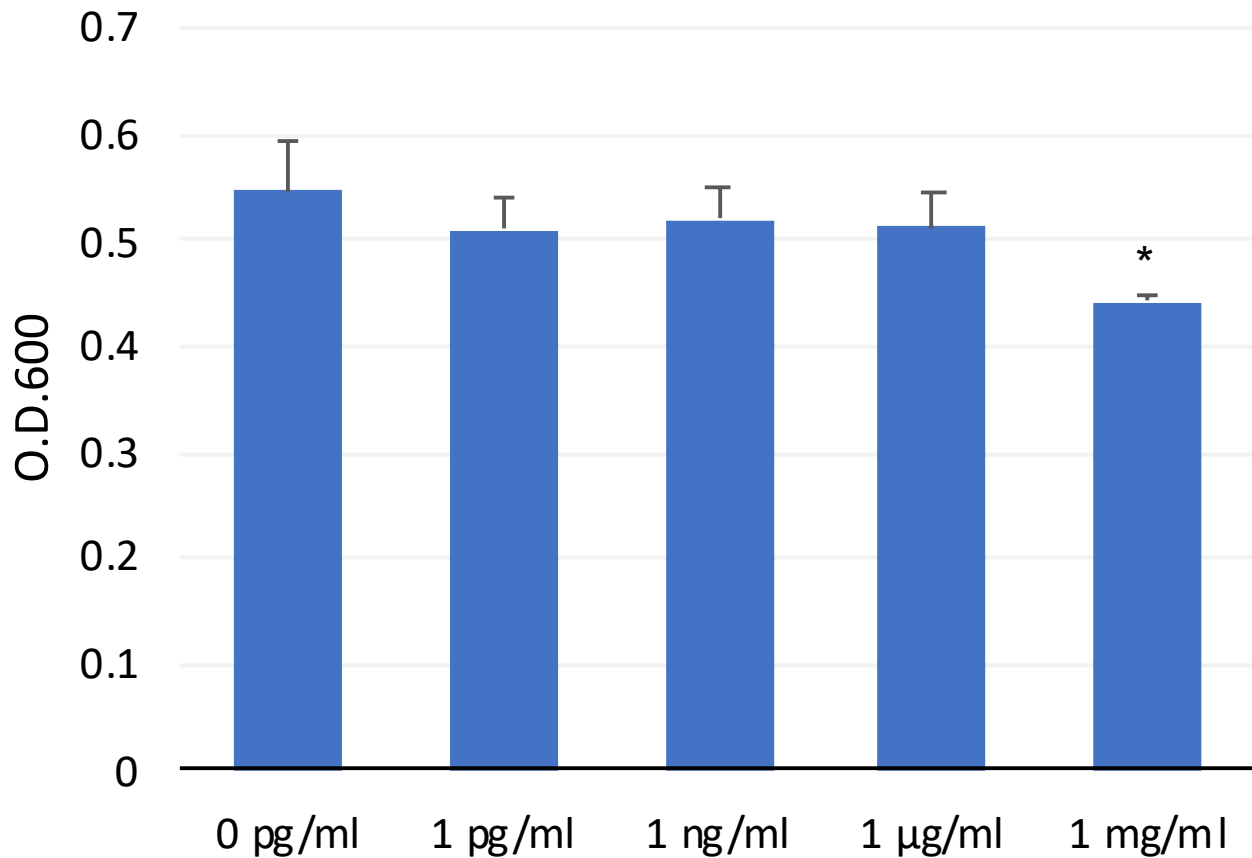

Supplement: Supplementary file 1 [file microorganisms-09-00593-s001.zip › 210206 Supplementary file /Fig.S2.pdf]

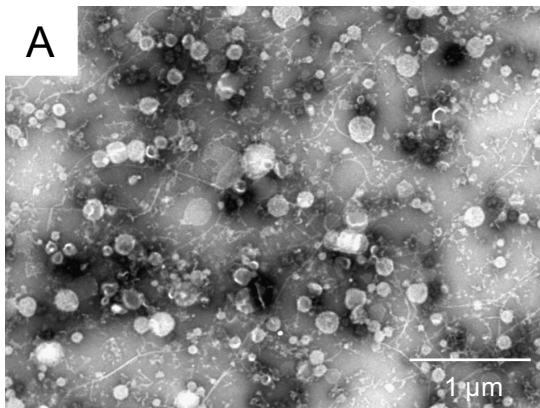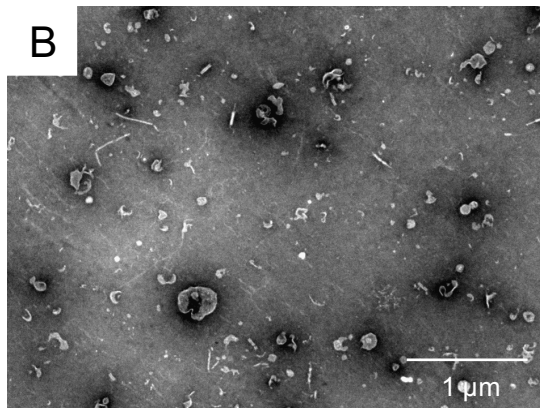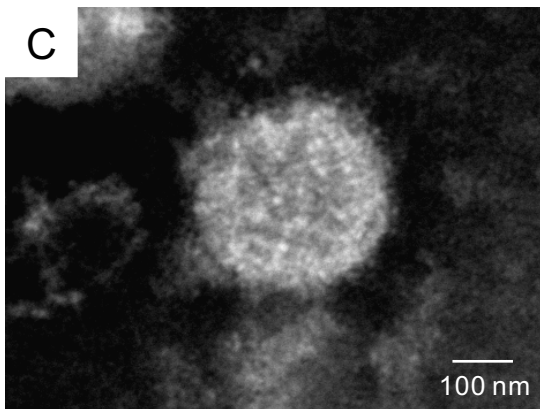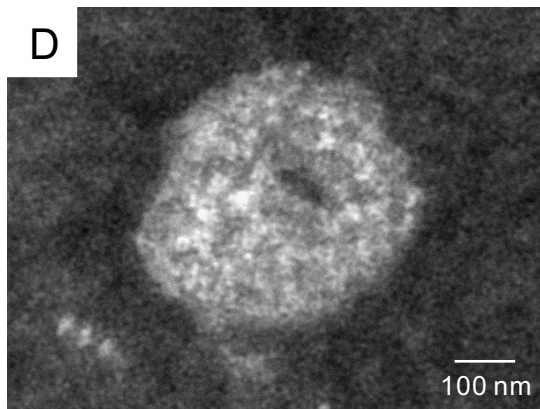

Supplement: Supplementary file 1 [file microorganisms-09-00593-s001.zip › 210206 Supplementary file /Fig.S1.pdf]
